# Assessing the Potential Association Between Microbes and Corrosion of Intra-Oral Metallic Alloy-Based Dental Appliances Through a Systematic Review of the Literature

**DOI:** 10.3389/fbioe.2021.631103

**Published:** 2021-03-15

**Authors:** Umarevathi Gopalakrishnan, A. Sumathi Felicita, Lodd Mahendra, Masroor Ahmed Kanji, Saranya Varadarajan, A. Thirumal Raj, Shaikh Mohammed Abdul Feroz, Deepak Mehta, Hosam Ali Baeshen, Shankargouda Patil

**Affiliations:** ^1^Department of Orthodontics, Sri Venkateswara Dental College and Hospital, Chennai, India; ^2^Department of Orthodontics, Saveetha Dental College, Saveetha Institute of Medical and Technical Sciences, Saveetha University, Chennai, India; ^3^Department of Prosthodontics, College of Applied Sciences, King Khalid University, Abha, Saudi Arabia; ^4^Department of Oral Pathology and Microbiology, Sri Venkateswara Dental College and Hospital, Chennai, India; ^5^Department of Prosthetic Dental Sciences, College of Dentistry, Jazan University, Jazan, Saudi Arabia; ^6^Department of Preventive and Restorative Dentistry, College of Dental Medicine, University of Sharjah, Sharjah, United Arab Emirates; ^7^Department of Orthodontics, College of Dentistry, King Abdulaziz University, Jeddah, Saudi Arabia; ^8^Division of Oral Pathology, Department of Maxillofacial Surgery and Diagnostic Sciences, College of Dentistry, Jazan University, Jazan, Saudi Arabia

**Keywords:** corrosion, metallic alloys, microorganism, oral, prosthesis

## Abstract

**Objective:** Systematic review assessing the association between oral microorganisms and corrosion of intra-oral metallic alloy-based dental appliances.

**Design:** PubMed, Scopus, and Web of Science were searched using keyword combinations such as microbes and oral and corrosion; microbes and dental and corrosion; microorganisms and oral and corrosion; microorganisms and dental and corrosion.

**Results:** Out of 141 articles, only 25 satisfied the selection criteria. *Lactobacillus reuteri, Streptococcus mutans, Streptococcus sanguis, Streptococcus mitis, Streptococcus sobrinus, Streptococcus salivariu*s, sulfate-reducing bacteria, sulfate oxidizing bacteria, Veilonella, Actinomyces, *Candida albicans* were found to have a potential association with corrosion of intraoral metallic alloys such as stainless steel, titanium, nickel, cobalt-chromium, neodymium-iron-boron magnets, zirconia, amalgam, copper aluminum, and precious metal alloys.

**Conclusion:** The included studies inferred an association between oral microorganisms and intra-oral metallic alloys-based dental appliances, although, it is vital to acknowledge that most studies in the review employed an *in-vitro* simulation of the intra-oral condition.

## Introduction

Metals in their pure or alloy forms are commonly used in dentistry despite the introduction of advanced materials like resins and ceramics, which can be largely attributed to the mechanical properties of metallic alloys (Upadhyay et al., 2006). The intra-oral environment has several factors that could predispose such metal alloy-based dental appliances to corrosion. These factors include varying temperature, oxygenation, mechanical forces, acidity, and alkalinity of external agents (foods, drugs), microorganisms, local anaerobic environments (e.g., subgingival). Some of the metals used in dentistry are amalgams of silver-tin, copper, noble metal alloys of gold and silver palladium, base metal alloys of nickel, cobalt, iron, and titanium alloys. Though most of the alloys are passivized and resistant to corrosion, the susceptibility still exists because of the predisposing factors in the oral environment (Bayramoglu et al., 2000; Karov and Hinberg, 2001). The clinical relevance of corrosion of dental appliances in the oral environment is due to some major clinical implications. The first is the potential toxic risk posed by the corrosion by-products. The second is that the corroded dental appliance could lose its functional integrity. The risk of allergy to the unbounded metal elements when released by corrosion should also be considered. A study by zora et al. suggested that corrosion products may pose a risk in immunologically susceptible patients (Venclíková et al., 2007).

The role of microorganisms in corrosion is extensively discussed in the sewage and pipeline industry, although the literature is relatively scanty when it comes to the biological environment, including the oral cavity (Mystkowska et al., 2018). Thus, the present systematic review was formulated to assess the association between microorganisms and corrosion of intra-oral dental appliances.

## Materials and Methods

**Figure 1:**
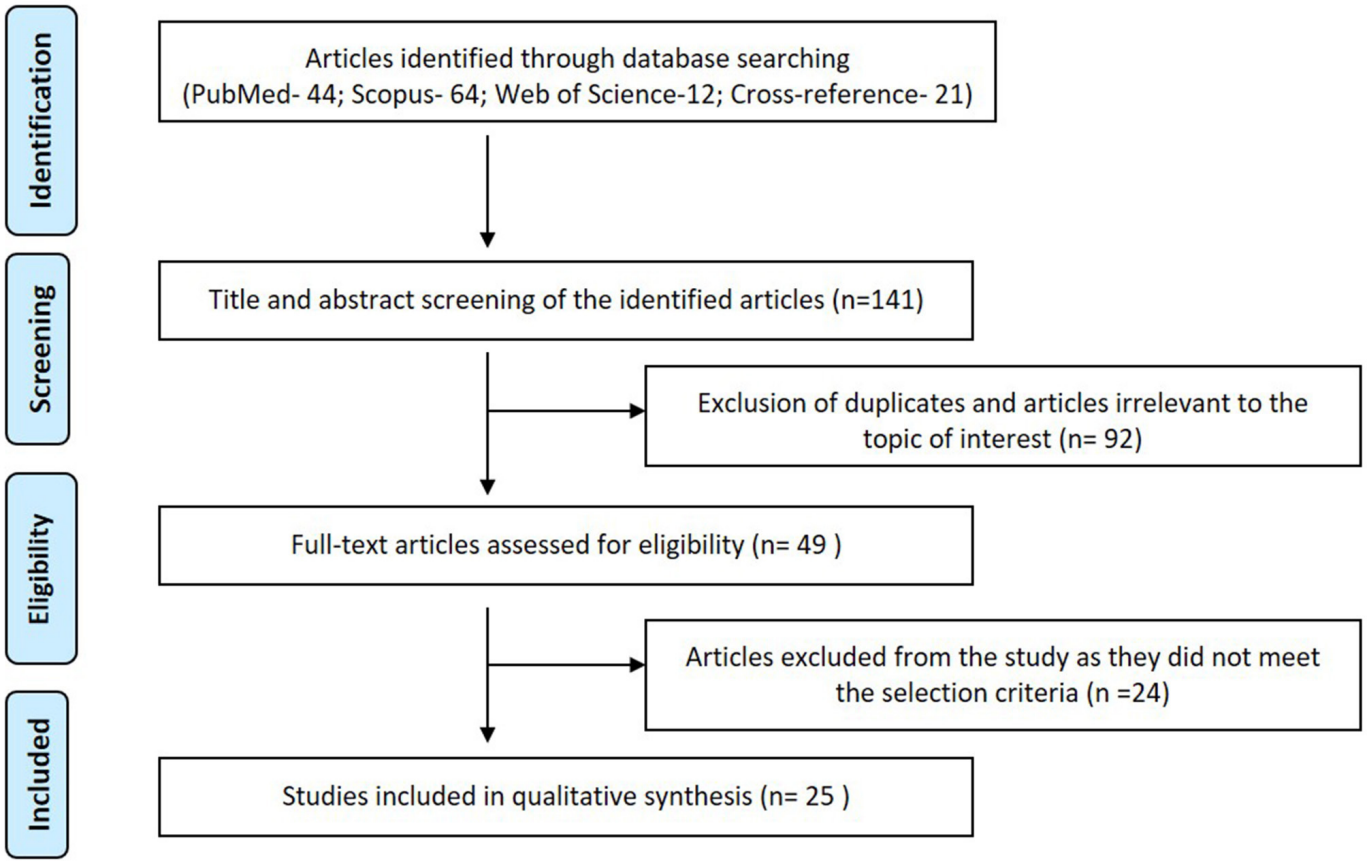
PRISMA flow diagram summarizing the study selection.

The present systematic review adhered strictly to the Preferred Reporting Items for Systematic Reviews and Meta-Analyses (PRISMA) statement (Moher et al., 2009; Hutton et al., 2015) (Figure 1).

### Inclusion Criteria

*In-vitro* studies in the English language assessing the potential effect of intra oral micro organisms on corrosion of metallic alloy-based dental appliances.

### Exclusion Criteria

*In-vivo* studies, reviews, letters, case reports/series, editorials. Articles not in the English language. Articles without sufficient details on either the microbe or the dental appliance, for *invivo* studies, due consideration to antibiotic use during sampling was checked. *In-vivo* studies were excluded as the research design could potentially play a major role in determining the final outcome. In addition, a prilimanary literature search revealed that at present, there are were no *in-vivo* studies which have assessed the effect of intra oral micro organisms on corrosion of metallic alloy-based dental appliances.

### Focus Question

What is the effect of an intra-oral microorganism on the corrosion of intraoral metallic alloy-based dental appliances? (population –metallic alloy-based dental appliances, intervention–intra oral microorganisms, comparator- metallic alloy-based dental appliances without oral microorganisms, outcome–corrosion).

### Search Strategy

PubMed, Scopus, and Web of Science were searched using various combinations of the following keywords: microbes and oral and corrosion; microbes and dental and corrosion; microorganisms and oral and corrosion; microorganisms and dental and corrosion. The identified articles were manually cross-referenced to identify further potential articles.

### Study Selection and Data Extraction

- Identified articles were screened for relevance to the topic and potential duplicates using their titles and abstracts.
- The full text of the screened articles was assessed using the selection criteria

Two reviewers (UG and SV) independently performed steps 1 and 2. Kappa coefficient (κ) was calculated to assess inter-observer reliability. Only studies satisfying the selection criteria were included in the qualitative analysis. Data including the study characteristics, design, assessment tools, the microbe, the metallic alloy assessed, results, and inference were extracted from these included articles. Due to the lack of a standard risk of bias tool for *in-vitro* studies, a customized risk of bias tool was formulated. The categorization in to high, medium, and low risk was based on Joanna Brigg's critical appraisal tool (The Joanna Briggs Institute, 2014; Normando et al., 2017).

## Results

### Study Selection

Hundred and forty-one articles (PubMed- 44; Scopus- 64; Web of Science-12; Cross-reference- 21) were identified in the search. Title and abstract screening led to the exclusion of 92 articles as they were either duplicate or lacked relevance to the topic of interest. Of the 49 articles subjected to full-text review, 24 articles were excluded as they did not fulfill the inclusion criteria (Supplementary Table 1). Only 25 articles met the eligibility criteria and were included in this review. Figure 1 summarizes the selection strategy employed in the qualitative analysis. Table 1 summarizes the data extracted from the studies included in the systematic review. Kappa coefficient (κ) for 1st and 2nd step of the review was 0.97 and 0.94, respectively indicating a good interreviewer reliability.

**Table 1:** Data extracted from the studies included in the systematic review.

**S. no**	**Author name/year of publication/country**	**Study design**	**Metallic alloy assessed**	**The diagnostic modality employed to assess corrosion**	**Microorganisms assessed**	**Inference**
1	Pavlic/2018/Croatia (Pavlic et al., 2019)	*In-vitro*	SUS, Ti mini implants	Surface roughness, microhardness by AFM and Vickers method	Probiotic bacteria *Lactobacillus reuteri*	Probiotics increase the surface roughness of Titanium and not stainless steel
2	Kameda/2019/Japan (Kameda et al., 2019)	*In vitro*	SUS and NiTi orthodontic wires	Surface roughness by laser confocal microscopy	*Streptococcus* (S) *mutans* and *S. sanguinis*.	Oral bacteria caused roughness in SUS wires
3	Cwalina/2017/Poland (Cwalina et al., 2017)	*In vitro*	NiTi, Ti alloy	A surface study by SEM, CLSM	Sulfur-oxidizing bacteria (SOB) and sulfate-reducing bacteria (SRB)	Both SOB and SRB colonize alloy surfaces and are capable of causing corrosion
4	Diaz/2017/Spain (Díaz et al., 2018)	*In vitro*	Ti alloy	A surface study by SEM	*Streptococcus mutans*	*Streptococcus mutans* negatively affected the corrosion resistance of titanium (augmented corrosion)
5	Lu/2017/China (Lu et al., 2017)	*In vitro*	NiCr, CoCr	A surface study by SEM	*Streptococcus mutans*	Presence of S. mutans in the solution reduced the corrosion rate of the alloys
6	Mystkowska/2016/Poland (Mystkowska, 2016)	*In vitro*	Co-Cr-Mo and Ti-6Al-4V	CSLM, XPS	*Desulfotomaculum nigrificans*	SRB caused significant corrosion of the alloy surface
7	Sridhar/2016/USA (Sridhar et al., 2016)	*In vitro*	Ti	A surface study by SEM	*Streptococcus mutans*	Bacteria (*S.mutans*) were able to create an acidic condition that triggered surface damage such as discoloration, rusting, and pitting.
8	Mystkowska/2015/Poland (Mystkowska et al., 2017)	*In vitro*	SUS	A surface study by CSLM	*Desulfotomaculum nigrificans*	*Desulfotomaculum nigrificans* caused corrosion of SUS
9	Pozhitkov/2015/USA (Pozhitkov et al., 2015)	*In vitro*	Ti implant	Electrochemical analysis	Diverse organisms of plaque (many species were found)	Microorganisms causes a significant amount of corrosion
10	Heggendorn/2015/Brazil (Heggendorn et al., 2015)	*In vitro*	SUS Endodontic files	A surface study by infinite focus alicona microscope	*Desulfovibrio desulfuricans* and *Desulfovibrio fairfieldensis*	*Desulfovibrio desulfuricans* and *Desulfovibrio fairfieldensis* caused biocorrosion of SUS files
11	Lucchetti/2015/Italy (Lucchetti et al., 2015)	*In vitro*	CoCr	Chemical analysis by atomic absorption spectrometer	*Eikenella corrodens*	No significant effect of bacteria on corrosion
12	Jorand/2015/France (Jorand et al., 2015)	*In vitro*	Ti	Surface study SEM and Raman spectroscopy	*Desulfovibrio fairfieldensis*	*Desulfovibrio fairfieldensis* is capable of causing corrosion
13	Kameda/2014/Japan (Kameda et al., 2014)	*In vitro*	SUS	Chemical analysis by plasma-optical emission spectrometer and CSLM	*Streptococcus mutans* and *Streptococcus sanguinis*	*Streptococcus mutans* and *Streptococcus sanguinis* did corrode orthodontic SUS appliances.
14	Fukushima/2014/Japan (Fukushima et al., 2014)	*In vitro*	Ti	Chemical analysis by coupled plasma-mass spectrometry	*Streptococcus mutans*	Bacteria induce corrosive properties of the titanium surface
15	Mabilleau/2006/France (Mabilleau et al., 2006)	*In vitro*	Ti	AFM and SEM	*Streptococcus mitis*.	*S.mitis* caused increased surface roughness of Ti
16	Laurent/2001/France (Laurent et al., 2001)	*In vitro*	Ni-Cr alloy and gold-based alloy	Electrochemical analysis and SEM	*Actinomyces viscosus*	Actinomyces viscosus caused corrosion of the concerned alloy
17	Vaidhyanadhan/1991/USA (Vaidyanathan et al., 1991)	*In vitro*	Five different alloys like gold, copper, silver, nickel	Visual examination of macrophotographs	*Actinomyces viscosus*	Actinomyces viscosus caused corrosion of alloys
18	Souza/2010/Portugal (Souza et al., 2010)	*In vitro*	Ti	Electrochemical tests to assess Ti	*Streptococcus mutans;* *Candida albicans*	The specified organisms lead to corrosion of Ti alloy
19	Maruthamuthu/2005/India (Maruthamuthu et al., 2005)	*In vitro*	NiTi, SUS	Corrosion potential by Polarization curves and electrochemical impedance spectroscopy of wires	Heterotrophic bacteria; Manganese oxidizing bacteria; Iron oxidizing bacteria; Acid-producing bacteria; Sulfate reducing bacteria.	Bacteria improves the corrosion resistance of NiTi (0.016) and SUS 26 gauge but slightly increases corrosion of SUS 0.016 wire
20	Célio G. Figueiredo-Pina/2018/Portugal (Figueiredo-Pina et al., 2019)	*In vitro*	Zirconia, Ti alloy	Electric Potential for corrosion current	*Streptococcus salivarius*	The titanium alloy corrosion activity during reciprocating sliding decreases when the bacteria species is present
21	Song-Mei Zhang/2013/China (Zhang et al., 2013)	*In vitro*	Ti	Surface roughness with SEM, electrochemical corrosion by impedence spectroscopy and electrochemical analysis by X-ray photoelectron spectroscopy	*Actinomyces naeslundii*	A. naeslundii can increase corrosion of titanium
22	Jui-Chung Chang/2003/USA (Chang et al., 2003)	*In vitro*	Pure Ti, Ti alloy, SUS,CoCr alloy, Ni-Cr alloy	Open circuit potential, potentiodynamic corrosion test, Stern-Geary corrosion test	*Streptococcus mutans*	Microbiology-related corrosion will occur due to the increased concentration of *S. mutans*.
23	Y. Oshida/2003/USA (Oshida et al., 2003)	*In vitro*	Pure Ti, Ti alloy, SUS, CoCr alloy, Ni-Cr alloy, Au-Ag alloy	Electric Potential for corrosion current	*Streptococcus mutans* and its products	The less noble materials (except CpTi grade II) showed their inferior corrosion resistance when they were exposed to media containing bacteria byproducts
24	L. Proenc/2015/Portugal (Proença et al., 2015)	*In vitro*	Ni–Cr–Mo alloy	Open circuit potential measurements, cyclic voltammetry, linear sweep voltammetry, as well as electronic microscopy coupled to electron diffraction spectroscopy	*Streptococcus sobrinus* and *Streptococcus mutans*	A 24 h immersion confirmed bio-corrosion of the alloy by S.mutans through the dissolution of Ni.
25	Adriana Cristina Zavanelli/2015/Brazil (Zavanelli et al., 2015)	*In vitro*	Amalgam and copper/aluminum alloy	Atomic absorption spectrophotometer	*Streptococcus mutans*	The S. mutans adhere to both amalgam and copper/aluminum alloy and cause corrosion

### Risk of Bias

Of the 25 studies included, 4 studies had a low risk of bias and 4 studies had a high risk of bias. The rest of the studies (*n* = 16) had moderate risk of bias. The summary of the risk of bias assessment is presented in Table 2.

**Table 2:** Summary of the risk of bias assessment of the studies included in the systematic review.

**S. no**	**Author name/year of publication/country**	**Control**	**Sample size**	**Description of strain used, method of procurement, culture specifications**	**Description of biomaterial tested, size, material composition clarity, surface details for texture**	**Methodology for assessment, equipment specifications, qualitative/quantitative assessment**	**Blinding of observer**	**Risk of bias**
1	Pavlic /2018/Croatia (Pavlic et al., 2019)	Y	Y	Y	Y	Y	N	Low
2	Kameda/2019/Japan (Kameda et al., 2019)	Y	N	N	Y	Y	N	Moderate
3	Cwalina/2017/Poland (Cwalina et al., 2017)	N	N	Y	Y	Y	N	Moderate
4	Diaz/2017/Spain (Díaz et al., 2018)	N	N	Y	Y	Y	N	Moderate
5	Lu/2017/China (Lu et al., 2017)	Y	N	Y	Y	Y	N	Low
6	Mystkowska/2016/Poland (Mystkowska, 2016)	Y	N	Y	Y	Y	N	Low
7	Sridhar/2016/USA (Sridhar et al., 2016)	N	N	N	N	Y	N	High
8	Mystkowska/2015/Poland (Mystkowska et al., 2017)	N	N	Y	Y	Y	N	Moderate
9	Pozhitkov/2015/USA (Pozhitkov et al., 2015)	Y	N	Y	Y	Y	N	Moderate
10	Heggendorn/2015/Brazil (Heggendorn et al., 2015)	Y	N	Y	Y	N	N	Moderate
11	Lucchetti/2015/Italy (Lucchetti et al., 2015)	Y	N	N	Y	Y	N	Moderate
12	Jorand/2015/France (Jorand et al., 2015)	Y	N	N	N	N	N	High
13	Kameda/2014/Japan (Kameda et al., 2014)	Y	N	N	Y	Y	N	Moderate
14	Fukushima/2014/Japan (Fukushima et al., 2014)	N	N	Y	Y	Y	N	Moderate
15	Mabilleau/2006/France (Mabilleau et al., 2006)	Y	N	Y	Y	Y	N	Low
16	Laurent/2001/France (Laurent et al., 2001)	N	Y	Y	Y	Y	N	Moderate
17	Vaidhyanadhan/1991/USA (Vaidyanathan et al., 1991)	N	N	N	Y	N	N	High
18	Souza/2010/Portugal (Souza et al., 2010)	N	N	Y	Y	Y	N	Moderate
19	Maruthamuthu/2005/India (Maruthamuthu et al., 2005)	N	N	N	N	N	N	High
20	Célio G. Figueiredo-Pina/2018/Portugal (Figueiredo-Pina et al., 2019)	N	N	Y	Y	Y	N	Moderate
21	Song-Mei Zhang/2013/China (Zhang et al., 2013)	Y	N	Y	Y	Y	N	Moderate
22	Jui-Chung Chang/2003/USA (Chang et al., 2003)	Y	N	Y	Y	Y	N	Moderate
23	Y. Oshida/2003/USA (Oshida et al., 2003)	Y	N	Y	Y	Y	N	Moderate
24	L. Proenc/2015/Portugal (Proença et al., 2015)	Y	N	Y	Y	Y	N	Moderate
25	Adriana Cristina Zavanelli/2015/Brazil (Zavanelli et al., 2015)	Y	N	N	Y	Y	N	Moderate

*Risk of bias categorized as high when the study reached up to 49% score yes, moderate when the study reached 50–69% score yes, and low when the study reached more than 70% score yes*.

### Study Characteristics

Of the 25 articles selected 5 were from the USA (Vaidyanathan et al., 1991; Chang et al., 2003; Oshida et al., 2003; Pozhitkov et al., 2015; Sridhar et al., 2016), 3 each were from Japan (Fukushima et al., 2014; Kameda et al., 2014, 2019), Portugal (Souza et al., 2010; Proença et al., 2015; Figueiredo-Pina et al., 2019), France (Laurent et al., 2001; Mabilleau et al., 2006; Jorand et al., 2015), Poland (Mystkowska, 2016; Cwalina et al., 2017; Mystkowska et al., 2017), 2 each from Brazil (Heggendorn et al., 2015; Zavanelli et al., 2015) and China (Zhang et al., 2013; Lu et al., 2017), 1 each from Croatia (Pavlic et al., 2019), Spain (Díaz et al., 2018), Italy (Lucchetti et al., 2015), and India (Maruthamuthu et al., 2005).

### Main Findings

Vital data including the assessed metallic alloy, the microorganisms, the methodology employed for detecting the microbe, and for assessing the corrosion, the statistical data, and the inferences drawn were extracted from all the included studies (Table 1). Titanium (Ti) was assessed in 16 studies (Oshida et al., 2003; Maruthamuthu et al., 2005; Mabilleau et al., 2006; Souza et al., 2010; Fukushima et al., 2014; Kameda et al., 2014; Jorand et al., 2015; Pozhitkov et al., 2015; Mystkowska, 2016; Sridhar et al., 2016; Díaz et al., 2018; Figueiredo-Pina et al., 2019), stainless steel (SUS) in five studies (Chang et al., 2003; Oshida et al., 2003; Maruthamuthu et al., 2005; Kameda et al., 2014; Heggendorn et al., 2015; Mystkowska et al., 2017), nickel (NiCr) and cobalt-chromium (CoCr) alloys in seven studies (Laurent et al., 2001; Chang et al., 2003; Oshida et al., 2003; Lucchetti et al., 2015; Proença et al., 2015; Mystkowska, 2016; Lu et al., 2017), neodymium-iron, zirconia (Figueiredo-Pina et al., 2019), amalgam and copper aluminum alloy (Zavanelli et al., 2015) and precious metal alloys (Vaidyanathan et al., 1991) were each assessed on one study. The bacteria that were studied include probiotic bacteria *Lactobacillus reuteri* (Pavlic et al., 2019), *Streptococcus (S.) mutans* (Chang et al., 2003; Oshida et al., 2003; Souza et al., 2010; Fukushima et al., 2014; Kameda et al., 2014, 2019; Zavanelli et al., 2015; Sridhar et al., 2016; Lu et al., 2017), *S.sanguis* (Kameda et al., 2014, 2019), *S.mitis* (Mabilleau et al., 2006), *S.obrinus* (Proença et al., 2015), *S.salivarius* (Figueiredo-Pina et al., 2019) sulfate-reducing bacteria (SRB) (Maruthamuthu et al., 2005; Heggendorn et al., 2015; Jorand et al., 2015; Mystkowska, 2016; Cwalina et al., 2017; Mystkowska et al., 2017), sulfate oxidizing bacteria (SOB) (Cwalina et al., 2017), *Actinomyces* (Vaidyanathan et al., 1991; Laurent et al., 2001; Zhang et al., 2013), *Eikenella* (Lucchetti et al., 2015), *Candida albicans* (Souza et al., 2010) and non-specific oral bacteria (Maruthamuthu et al., 2005; Pozhitkov et al., 2015). The corrosion property was studied with scanning electron microscope (SEM) (Laurent et al., 2001; Mabilleau et al., 2006; Zhang et al., 2013; Jorand et al., 2015; Proença et al., 2015; Sridhar et al., 2016; Cwalina et al., 2017; Lu et al., 2017), confocal laser scanning microscopy (CSLM) (Kameda et al., 2014, 2019; Mystkowska, 2016; Cwalina et al., 2017; Mystkowska et al., 2017), microhardness with atomic force microscopy (AFM) (Mabilleau et al., 2006; Pavlic et al., 2019), atomic absorption spectrophotometry (Zavanelli et al., 2015), mass spectroscopy (Lucchetti et al., 2015; Pozhitkov et al., 2015), corrosion potential measurement (Chang et al., 2003; Oshida et al., 2003; Maruthamuthu et al., 2005; Proença et al., 2015; Figueiredo-Pina et al., 2019), impedance spectroscopy (Maruthamuthu et al., 2005; Zhang et al., 2013), and electrochemical analysis (Souza et al., 2010). Only one study by Vaidhyanadhan et al. (26) used visual examination by macro photography to assess the corrosion. Probiotics-like Lactobacillus Reuteri (Pavlic et al., 2019) increases the surface roughness of Ti mini-implants while less effect is seen on SUS. In a study by Kameda et al. (2019), oral bacteria like *S.mutans* and *S.sanguinis* were shown to corrode stainless steel orthodontic wires. *S.mutans* has been shown to increase the corrosion of both SUS and Ti alloys (Chang et al., 2003; Oshida et al., 2003; Souza et al., 2010; Fukushima et al., 2014; Kameda et al., 2014, 2019; Proença et al., 2015; Zavanelli et al., 2015; Sridhar et al., 2016; Díaz et al., 2018). *S.mitis* has also been shown to be corrosive toward Ti alloy (Mabilleau et al., 2006). Sulfate-reducing bacteria (Heggendorn et al., 2015; Jorand et al., 2015; Mystkowska, 2016; Cwalina et al., 2017; Mystkowska et al., 2017) have been shown to corrode SUS and Ti alloys. *Actinomyces* (Vaidyanathan et al., 1991; Laurent et al., 2001; Zhang et al., 2013) caused corrosion of NiCr and other precious metal alloys. *Eikenella* (Lucchetti et al., 2015) did not show any association with the corrosion of metals. *Candida albicans* has been shown to have a corrosive influence on Ti (Souza et al., 2010). Maruthamuthu et al. (2005) reported that bacteria improve the corrosion resistance of NiTi (0.016) and SUS 26 gauge but slightly increases corrosion of SUS 0.016 wire. Figueiredo-Pina et al. (2019) reported that the presence of *S.salivarious* in the lubricant reduces the corrosion wear of Ti. Table 3 summarizes the effect of oral-microorganisms on the corrosion of metal alloys.

**Table 3:** Effect of oral microorganism on the corrosion of metal alloy in the included studies.

**Microorganism**	**Metal alloy**	**Effect on corrosion**
*Lactobacillus reuteri*	SUS, Ti	Augmented corrosion
*Streptococcus mutans*	SUS, Ni-Ti, Ti, Au-Ag, Co-Cr, Ni-Cr-Mo	Augmented corrosion
	Ni-Cr, Co-Cr	Inhibited corrosion
	Ni-Ti, Amalgam and Cu/Al	No effect on corrosion
*Streptococcus sanguinis*	SUS	Augmented corrosion
	Ni-Ti	No effect on corrosion
*Sulfur-oxidizing bacteria*	Ni-Ti	Augmented corrosion
*Sulfate-reducing bacteria*	Ni-Ti	Augmented corrosion
*Desulfotomaculum nigrificans*	Co-Cr-Mo, Ti-6Al-4V, SUS	Augmented corrosion
*Desulfovibrio desulfuricans*	SUS	Augmented corrosion
*Desulfovibrio fairfieldensis*	SUS, Ti	Augmented corrosion
*Eikenella corrodens*	CoCr	No effect on corrosion
*Streptococcus mitis*	Ti	Augmented corrosion
*Actinomyces viscosus*	Ni-Cr, Au, Cu, Ag, Ni	Augmented corrosion
*Candida albicans*	Ti	Augmented corrosion
*Heterotrophic bacteria;* *Manganese oxidizing bacteria;* * Iron oxidizing bacteria;* * Acid-producing bacteria;* * Sulphate reducing bacteria*.	NiTi, SUS	Augmented corrosion
*Streptococcus salivarius*	Ti	Inhibited corrosion
	Zr	No effect on corrosion
*Actinomyces naeslundii*	Ti	Augmented corrosion
*Streptococcus sobrinus*	Ni– Cr–Mo	No effect on corrosion

## Discussion

Microbial Corrosion of metal is induced by activities of microorganisms like bacteria, fungi, and algae (Wilson et al., 1997; Daubert et al., 2018). The bacteria more commonly attributed to corrosion are SRB, SOB, iron-oxidizing/ reducing bacteria, manganese-oxidizing bacteria, Pseudomonas, bacteria secreting organic acids, and slime. Among the fungi, *Cladosporium, Aspergillus, Penicillium, and Paecilomyces* (Iverson, 1987), and *Candida albicans* (Souza et al., 2010) are associated with metallic alloy corrosion. Bluegreen algae and a species of red algae (*Graciollasia sp*.) are the algae associated with corrosion (Iverson, 1987). In the present article, the published literature was reviewed to assess the association between oral microbes and corrosion in intra-oral dental materials.

It was observed that various species like *Streptococcus, Actinomyces, Veilonella*, SRB, SOB, were reported to cause corrosion intraorally. There can be two categories of microbial corrosion based on the involvement of oxygen, anaerobic, and aerobic corrosion. SRB is a classic example of anaerobic corrosion while SOB is a prime example of aerobic corrosion. The basic process of corrosion involves a flow of electricity between certain areas of a metal surface through a solution that can conduct an electric current. Organisms like SOB secrete organic acids as part of their fermentation process which in turn stimulates anodic reactions. Sulphuric acid produced by SOB reduces the pH which in turn favors the growth of iron and manganese-oxidizing bacteria. These microbes oxidize manganese and iron metal alloys and cause their corrosion (Maruthamuthu et al., 2005). Pavlic et al. (2019), Sridhar et al. (2016), Pozhitkov et al. (2015), and Vaidyanathan et al. (1991) reported that a difference in pH could have contributed to the microbial corrosion. Literature suggests that the lowering pH, although may not corrode as the pH does not reach the depassivation point, it is plausible that it may favor the process (Nash and Kelly, 1993; Schiff et al., 2002). Mabilleau et al. (2006) suggested that S.mitis releases lactic acid in the microenvironment and it is likely that this compound is the main candidate to explain Ti corrosion. Some organisms stimulate cathodic reactions by consuming hydrogen. Sulfate-reducing bacteria (SRB) consume hydrogen through hydrogenase enzymes thereby depolarizing the cathode enhancing the process of corrosion (Mystkowska, 2016). SRB also utilize lactate produced by other bacteria in the biofilm as a carbon source and reduce sulfate to sulfide. Sulfide combines with iron in SUS alloys to form ferrous sulfide as the corrosion product. Cwalina et al. (2017) found that both groups of bacteria of sulfur cycle, SRB, and SOB colonize NiTi and Ti alloys, with a lower pH favoring the growth of SRB and causing further corrosion. Both SRB and SOB are capable of corroding NiTi and Ti alloys even though Kameda et al. (2014) found a higher degree of corrosion in SUS and none in Ti. This in turn could be because Ti is more resistant to corrosion by electric current.

Corrosion cells also occur when two areas are in contact with different concentrations of the same solution, like a difference in concentration of oxygen. The less-aerated zone acts as an anode, which undergoes corrosion. One of the factors causing such oxygenation difference is the heterogeneous layer of a biofilm with bacteria like *Streptococcus mutans* which use oxygen and create a difference in degrees of oxygen concentration based on their presence or absence in the biofilm (Alasvand Zarasvand and Rai, 2014). This is the main mechanism behind the corrosion of *S.mutans*. Fukushima et al. (2014) also suggested a similar mechanism in their study. *Actinomyces viscosus* consumes oxygen and shifts the anodic curve toward more negative potentials causing corrosion of metals (Laurent et al., 2001). In addition to other reasons, Díaz et al. (2018) has suggested that surface roughness promotes the corrosion of the Ti surface by *S.mutans* by creating retentive areas for the bacteria.

There were few contrary findings regarding microbial corrosion. Lu et al. (2017) stated that *S. mutans* formed a biofilm on the metal surface which enhances corrosion resistance by creating physical barriers that prevented oxygen interactions with the metal surfaces. Lucchetti et al. (2015) too found no significant effect of bacteria like *Eikenella corrodens* on corrosion of metal alloys. A study by Maruthamuthu et al. (2005) has shown that passivity and corrosion resistance of some SUS and NiTi was improved by bacteria whereas some SUS was shown to decrease. In a study by Liu et al. (2018) found that rapid electrochemical anodization treatment used on Ti2448 alloys increased their biocorrosion resistance. Regarding the prevention against microbial corrosion, Liu et al. (2018) suggested that a new beta-type Ti alloy with a hybrid oxide layer produced by the electrochemical anodization treatment provided better protection against corrosion by microorganisms by lowering the anodic and cathodic current densities. Jorand et al. (2015) showed that the SRB is resistant to ampicillin therapy which might sound that fighting corrosion against these organisms might be difficult. Microbial corrosion needs higher attention in dentistry as more evidence is gathered regarding their role in inducing intra-oral corrosion of alloys. The present systematic review provides insight into the various microorganisms implicated in causing corrosion of intraoral metallic alloy-based dental appliances. Also, the various mechanisms for a microbe induced metallic alloy corrosion are elaborated. Out of the 25 articles reviewed, 23 articles suggested that microorganisms are capable of causing corrosion while 2 articles (Maruthamuthu et al., 2005; Lu et al., 2017) suggested that they protect against corrosion and one suggested no significant effect (Lucchetti et al., 2015).

Although all the 25 studies had assessed the role of microorganisms in corrosion of dental appliance, most were *in-vitro* studies simulating the intra-oral conditions. Also, there were several variables including methodology used to assess the corrosion, the research design (*in-vitro/in-vivo* microenvironment) employed, the microbe and the metal/alloys assessed which led to large-scale heterogeneity in the collected data. In addition, in studies like Pozhitkov et al., the results did not specify the microorganisms responsible for the corrosion (Pozhitkov et al., 2015). Given the significant number of variables in the included studies and the lack of specificity in reporting the the causative microbe, a quantitative analysis was not possible.

## Conclusion

The review identified several microorganisms to be closely associated with corrosion of intraoral metallic alloy-based dental appliance. Despite the association, it is vital to acknowledge that most of the included studies were based on *in-vitro* models. Thus, large-scale multi-center prospective clinical studies with a homogenous research design are required to validate the findings of the present systematic review.

## Data Availability Statement

The original contributions presented in the study are included in the article/Supplementary Material, further inquiries can be directed to the corresponding author/s.

## Author Contributions

UG, AF, LM, SP, SV, and AR contributed to the conception of the work, data acquisition, analysis, drafting the work, and final approval of the version to be published. MK, SF, DM, and HB contributed to the interpretation of data, revising it critically for important intellectual content, and final approval of the version to be published. All authors agree to be accountable for all aspects of the work ensuring that questions related to the accuracy or integrity of any part of the work are appropriately investigated and resolved.

## Conflict of Interest

The authors declare that the research was conducted in the absence of any commercial or financial relationships that could be construed as a potential conflict of interest.
