# Supplementary material for: Assessing the Potential Association Between Microbes and Corrosion of Intra-Oral Metallic Alloy-Based Dental Appliances Through a Systematic Review of the Literature
Source: Front Bioeng Biotechnol. 2021 Mar 15;9:631103. doi: 10.3389/fbioe.2021.631103 (PMC8005604; doi:10.3389/fbioe.2021.631103)
Supplement: Supplementary file 1 [file Table_1.DOCX]

**Supplementary table 1:** Summary of the articles excluded post-full text evaluation and the reasons for exclusion (n=24).

| **Reference** | **Author (Year)** | **Reasons for Exclusion** |
| --- | --- | --- |
|  | Zhou W et al (2019) | 1 |
|  | Li et al (2019) | 2 |
|  | Mombelli (2018) | 1 |
|  | Schliephake (2018) | 3 |
|  | Sridhar (2018) | 3 |
|  | Souza (2019) | 2 |
|  | Harada(2018) | 3 |
|  | Resende (2018) | 3 |
|  | Grippo(2018) | 3 |
|  | Edelhoff(2018) | 3 |
|  | Ottria (2018) | 3 |
|  | Divakar(2018) | 3 |
|  | Bhasin(2017) | 3 |
|  | Moorman (2017) | 3 |
|  | Machado (2017) | 3 |
|  | Klinger-Strobel (2016) | 3 |
|  | Niu (2016) | 4 |
|  | Ma (2016) | 4 |
|  | Jia (2016) | 3 |
|  | Barão(2015) | 2 |
|  | Kois (2015) | 3 |
|  | Jorand(2015) | 2 |
|  | Costa(2015) | 3 |
|  | Inger (2014) | 3 |

Legend: (1) corrosion study does not involve microorganisms (2) corrosion was not studied (3) not relevant to corrosion or microorganisms (4) not relevant intraorally

**References:**

1. Zhou W, Peng X, Zhou X, Li M, Ren B, Cheng L. Influence of bio-aging on corrosion behavior of different implant materials. Clin Implant Dent Relat Res. 2019 Dec;21(6):1225-1234. doi: 10.1111/cid.12865. Epub 2019 Nov 15. PMID: 31729828.
2. Li P, Zhang W, Dai J, Xepapadeas AB, Schweizer E, Alexander D, Scheideler L, Zhou C, Zhang H, Wan G, Geis-Gerstorfer J. Investigation of zinc‑copper alloys as potential materials for craniomaxillofacial osteosynthesis implants. Mater Sci Eng C Mater Biol Appl. 2019 Oct;103:109826. doi: 10.1016/j.msec.2019.109826. Epub 2019 May 30. PMID: 31349503.
3. Mombelli A, Hashim D, Cionca N. What is the impact of titanium particles and biocorrosion on implant survival and complications? A critical review. Clin Oral Implants Res. 2018 Oct;29 Suppl 18:37-53. doi: 10.1111/clr.13305. PMID: 30306693.
4. Schliephake H, Sicilia A, Nawas BA, Donos N, Gruber R, Jepsen S, Milinkovic I, Mombelli A, Navarro JM, Quirynen M, Rocchietta I, Schiødt M, Schou S, Stähli A, Stavropoulos A. Drugs and diseases: Summary and consensus statements of group 1. The 5^th^ EAO Consensus Conference 2018. Clin Oral Implants Res. 2018 Oct;29 Suppl 18:93-99. doi: 10.1111/clr.13270. Erratum in: Clin Oral Implants Res. 2020 Feb;31(2):201. PMID: 30306683.
5. Sridhar S, Wang F, Wilson TG Jr, Valderrama P, Palmer K, Rodrigues DC. Multifaceted roles of environmental factors toward dental implant performance: Observations from clinical retrievals and in vitro testing. Dent Mater. 2018 Nov;34(11):e265-e279. doi: 10.1016/j.dental.2018.08.299. Epub 2018 Sep 13. PMID: 30220507.
6. Souza JGS, Cordeiro JM, Lima CV, Barão VAR. Citric acid reduces oral biofilm and influences the electrochemical behavior of titanium: An in situ and in vitro study. J Periodontol. 2019 Feb;90(2):149-158. doi: 10.1002/JPER.18-0178. Epub 2018 Sep 11. PMID: 30088827.
7. Harada LK, Silva EC, Campos WF, Del Fiol FS, Vila M, Dąbrowska K, Krylov VN, Balcão VM. Biotechnological applications of bacteriophages: State of the art. Microbiol Res. 2018 Jul-Aug;212-213:38-58. doi: 10.1016/j.micres.2018.04.007. Epub 2018 Apr 30. PMID: 29853167.
8. Resende TH, Reis KR, Schlichting LH, Magne P. Ultrathin CAD-CAM Ceramic Occlusal Veneers and Anterior Bilaminar Veneers for the Treatment of Moderate Dental Biocorrosion: A 1.5-Year Follow-Up. Oper Dent. 2018 Jul/Aug;43(4):337-346. doi: 10.2341/17-007-T. Epub 2018 Mar 27. PMID: 29584553.
9. Grippo JO, Coleman TA, Messina AM, Oh DS. A literature review and hypothesis for the etiologies of cervical and root caries. J Esthet Restor Dent. 2018 May;30(3):187-192. doi: 10.1111/jerd.12365. Epub 2018 Jan 18. PMID: 29349909.
10. Edelhoff D, Ahlers MO. Occlusal onlays as a modern treatment concept for the reconstruction of severely worn occlusal surfaces. Quintessence Int. 2018;49(7):521-533. doi: 10.3290/j.qi.a40482. PMID: 29881829.
11. Ottria L, Lauritano D, Andreasi Bassi M, Palmieri A, Candotto V, Tagliabue A, Tettamanti L. Mechanical, chemical and biological aspects of titanium and titanium alloys in implant dentistry. J Biol Regul Homeost Agents. 2018 Jan-Feb;32(2 Suppl. 1):81-90. PMID: 29460522.
12. Divakar DD, Jastaniyah NT, Altamimi HG, Alnakhli YO, Muzaheed, Alkheraif AA, Haleem S. Enhanced antimicrobial activity of naturally derived bioactive molecule chitosan conjugated silver nanoparticle against dental implant pathogens. Int J Biol Macromol. 2018 Mar;108:790-797. doi: 10.1016/j.ijbiomac.2017.10.166. Epub 2017 Nov 1. PMID: 29102795.
13. Bhasin V, Pustake SJ, Joshi V, Tiwari A, Bhasin M, Punia RS. Assessment of Changes in Nickel and Chromium Levels in the Gingival Crevicular Fluid during Fixed Orthodontic Treatment. J Contemp Dent Pract. 2017 Aug 1;18(8):675-678. doi: 10.5005/jp-journals-10024-2105. PMID: 28816188.
14. Moorman E, Montazeri N, Jaykus LA. Efficacy of Neutral Electrolyzed Water for Inactivation of Human Norovirus. Appl Environ Microbiol. 2017 Aug 1;83(16):e00653-17. doi: 10.1128/AEM.00653-17. PMID: 28600317; PMCID: PMC5541222.
15. Machado AC, Soares CJ, Reis BR, Bicalho AA, Raposo L, Soares PV. Stress-strain Analysis of Premolars With Non-carious Cervical Lesions: Influence of Restorative Material, Loading Direction and Mechanical Fatigue. Oper Dent. 2017 May/Jun;42(3):253-265. doi: 10.2341/14-195-L. Epub 2017 Mar 13. PMID: 28467256.
16. Klinger-Strobel M, Makarewicz O, Pletz MW, Stallmach A, Lautenschläger C. TiO_2_-containing and ZnO-containing borosilicate glass-a novel thin glass with exceptional antibiofilm performances to prevent microfouling. J Mater Sci Mater Med. 2016 Dec;27(12):175. doi: 10.1007/s10856-016-5792-4. Epub 2016 Oct 17. PMID: 27752973.
17. Niu J, Tang Z, Huang H, Pei J, Zhang H, Yuan G, Ding W. Research on a Zn-Cu alloy as a biodegradable material for potential vascular stents application. Mater Sci Eng C Mater Biol Appl. 2016 Dec 1;69:407-13. doi: 10.1016/j.msec.2016.06.082. Epub 2016 Jun 27. PMID: 27612729.
18. Ma Z, Li M, Liu R, Ren L, Zhang Y, Pan H, Zhao Y, Yang K. In vitro study on an antibacterial Ti-5Cu alloy for medical application. J Mater Sci Mater Med. 2016 May;27(5):91. doi: 10.1007/s10856-016-5698-1. Epub 2016 Mar 14. PMID: 26975748.
19. Jia Z, Xiu P, Li M, Xu X, Shi Y, Cheng Y, Wei S, Zheng Y, Xi T, Cai H, Liu Z. Bioinspired anchoring AgNPs onto micro-nanoporous TiO2 orthopedic coatings: Trap-killing of bacteria, surface-regulated osteoblast functions and host responses. Biomaterials. 2016 Jan;75:203-222. doi: 10.1016/j.biomaterials.2015.10.035. Epub 2015 Oct 23. PMID: 26513414.
20. Barão VA, Ricomini-Filho AP, Faverani LP, Del Bel Cury AA, Sukotjo C, Monteiro DR, Yuan JC, Mathew MT, do Amaral RC, Mesquita MF, da Silva WJ, Assunção WG. The role of nicotine, cotinine and caffeine on the electrochemical behavior and bacterial colonization to cp-Ti. Mater Sci Eng C Mater Biol Appl. 2015 Nov 1;56:114-24. doi: 10.1016/j.msec.2015.06.026. Epub 2015 Jun 14. PMID: 26249572.
21. Kois DE, Kois JC. Comprehensive Risk-Based Diagnostically Driven Treatment Planning: Developing Sequentially Generated Treatment. Dent Clin North Am. 2015 Jul;59(3):593-608. doi: 10.1016/j.cden.2015.03.001. Epub 2015 Apr 18. PMID: 26140967.
22. Jorand FP, Debuy S, Kamagate SF, Engels-Deutsch M. Evaluation of a biofilm formation by Desulfovibrio fairfieldensis on titanium implants. Lett Appl Microbiol. 2015 Mar;60(3):279-87. doi: 10.1111/lam.12370. Epub 2015 Jan 7. PMID: 25431313.
23. Costa SA, Paula OF, Silva CR, Leão MV, Santos SS. Stability of antimicrobial activity of peracetic acid solutions used in the final disinfection process. Braz Oral Res. 2015;29:S1806-83242015000100239. doi: 10.1590/1807-3107BOR-2015.vol29.0038. Epub 2015 Feb 11. PMID: 25715037.
24. Inger M, Bennani V, Farella M, Bennani F, Cannon RD. Efficacy of air/water syringe tip sterilization. Aust Dent J. 2014 Mar;59(1):87-92. doi: 10.1111/adj.12146. Epub 2014 Feb 4. PMID: 24494727.
